# Fluid resuscitation in trauma: what are the best strategies and fluids?

**DOI:** 10.1186/s12245-019-0253-8

**Published:** 2019-12-04

**Authors:** G. H. Ramesh, J. C. Uma, Sheerin Farhath

**Affiliations:** 1 Emergency Department BMC & RI. Victoria Hospital, City Market, Bengaluru, Karnataka 560002 India; 2 K.C.G Hospital 89, 5th Cross Rd, Behind Police Station, Malleshwaram, Bengaluru, Karnataka 560003 India; 3 Columbia Asia Hospital Yeshwanthpur 26/4, Brigade Gateway Malleshwaram West Beside Metro Cash and Carry West, Yeswanthpur, Bengaluru, Karnataka 560055 India

**Keywords:** Fluid resuscitation, Trauma, Delayed resuscitation, Permissive hypotension, Prehospital care, Colloid, Crystalloid

## Abstract

**Background:**

Traumatic injuries pose a global health problem and account for about 10% global burden of disease. Among injured patients, the major cause of potentially preventable death is uncontrolled post-traumatic hemorrhage.

**Main body:**

This review discusses the role of prehospital trauma care in low-resource/remote settings, goals, principles and evolving strategies of fluid resuscitation, ideal resuscitation fluid, and post-resuscitation fluid management. Management of fluid resuscitation in few special groups is also discussed.

**Conclusions:**

Prehospital trauma care systems reduce mortality in low-resource/remote settings. Delayed resuscitation seems a better option when transport time to definitive care is shorter whereas goal-directed resuscitation with low-volume crystalloid seems a better option if transport time is longer. Few general recommendations regarding the choice of fluid are provided. Adhering to evidence-based clinical practice guidelines and local modifications based on patient population, available resources, and expertise will improve patient outcomes.

## Introduction

Traumatic injuries account for nearly 10% of the global burden of disease [1]. The major cause of potentially preventable death among injured patients is uncontrolled post-traumatic hemorrhage [2]. In trauma patients, fluid resuscitation helps restore lost blood volume, regain tissue perfusion, and reduce mortality.

This review discusses the role of prehospital trauma care (PTC) in low-resource/remote settings; goals, principles and evolving strategies of fluid resuscitation, particularly those before blood products are available; and post-resuscitation fluid management. Management of fluid resuscitation in some special groups is also discussed.

## Prehospital trauma care

PTC aims at the stoppage of bleeding to minimize further blood loss, initial resuscitation to maintain mental status and peripheral pulses, and fast transportation to hospital for definitive treatment [3]. Prehospital intravenous fluid administration decreases mortality in trauma patients, especially in major injuries and rural settings when prehospital transport time (PTT) is long [4–7].

## Goals and principles of fluid resuscitation

The goals of fluid resuscitation include controlling bleeding, restoring lost blood volume, and regaining tissue perfusion and organ function. Different target systolic blood pressure (SBP) values may be considered for different traumas: 60–70 mmHg for penetrating trauma; 80–90 mmHg for blunt trauma without traumatic brain injury (TBI); 100–110 mmHg for blunt trauma with TBI [8]. However, as clinical scenarios are complex and variable, adhering to evidence-based clinical practice guidelines and adapting according to local treatment and patient condition is likely to improve patient outcomes [2].

Fluid administration is beneficial only if it increases the stroke volume (SV) and thereby, the cardiac output. Patients are considered fluid responsive if SV increases by at least 10% after a fluid challenge of 500 mL of crystalloid [9]. Pulse pressure variation, passive leg raising test, and SV variation are some reliable markers for fluid responsiveness.

## Evolving strategies of fluid resuscitation

Resuscitation strategies are based on volume, rate, and time of fluid administration. Earlier, immediate aggressive fluid resuscitation in trauma patients was the standard approach to restore circulating volume and maintain organ perfusion. However, it may dislodge soft clots and cause dilutional coagulopathy thereby increasing hemorrhage and mortality [3]. Treating trauma patients with large crystalloid volumes leads to resuscitation injury, gastrointestinal and cardiac complications, increased extremity compartment pressures, coagulation disturbances, electrolyte imbalance, hypothermia, and abdominal compartment syndrome [3].

Two strategies were proposed to avoid clot disruption and dilutional coagulopathy: delayed resuscitation strategy where fluid is given after bleeding is controlled and permissive hypotension strategy, where fluid is given to increase SBP without reaching normotension. In penetrating trauma patients with hypotension (prehospital SBP < 90 mmHg), delayed resuscitation shows better survival rates compared to immediate resuscitation [10]. Increased mortality is seen with increased in-field procedures [11, 12], supporting “scoop and run” and delayed fluid resuscitation techniques. However, when PTT is long, simple life support measures reduce mortality in severely injured patients [4, 5] even when conducted in suburban and remote locations with long PTT [13].

The low volume fluid resuscitation during permissive hypotension maintains low tissue perfusion but is adequate for short periods. Permissive hypotension is achieved by goal-directed resuscitation [SBP/mean arterial pressure (MAP) is targeted based on patient physiology] or controlled resuscitation (predetermined fixed rates are infused such that normotension is not achieved) [3, 14]. In animal studies, controlled resuscitation of 60–80 mL/kg/h usually maintains SBP of 80–90 mmHg (MAP of 40–60 mmHg) in hemorrhagic shock patients [14].

Permissive hypotension is associated with decreased blood loss, intra-abdominal bleeding, risk of intra-abdominal hypertension, acidemia, hemodilution, thrombocytopenia, coagulopathy, apoptotic cell death, tissue injury, sepsis, volumes of crystalloid administration needed, and blood product utilization, and improved organ perfusion and survival [14, 15]. However, prolonged (8 h) hypotension (SBP < 65 mmHg or MAP around 65 mmHg) has been shown to increase metabolic stress, tissue hypoxia, and mortality in animal studies [14].

International guidelines recommend restrictive volume replacement approach to achieve target blood pressure (BP) until bleeding is controlled [2]. Penetrating trauma patients have better outcomes with SBP of 60–70 mmHg. In blunt trauma, higher SBP of 80–90 mmHg is permitted but slower infusions are preferred over large boluses [15]. In patients with traumatic hemorrhagic shock, permissive hypotension is safe and feasible, and reduces mortality [16]. However, large crystalloid volumes are not advisable and should be cautiously administered.

Mortality is increased in patients with head injury and low cerebral perfusion pressures [17]. Small aliquots of fluid (100–200 mL) should be administered to maintain SBP > 90 mmHg [18] or MAP > 80 mmHg [15]. Permissive hypotension is contraindicated in TBI [18].

When PTT to definitive care is shorter (< 10–15 min) and patients are well-selected, delayed resuscitation seems a good option [13, 14]. Advanced life support interventions provide no added benefit and may delay the time to definitive care [19]. When PTT is longer than 10–15 min, mortality is increased and goal-directed resuscitation with low-volume crystalloid and basic/advanced life support interventions are better [4, 5, 13, 14]. As clinical scenarios are complex and variable, adhering to evidence-based clinical practice guidelines and individualizing the patient’s SBP/MAP goals are important.

In severely injured patients, the lethal triad of hypothermia, acidosis, and coagulopathy exacerbates hemorrhage. Damage control resuscitation combats this and comprises of permissive hypotension, hemostatic resuscitation, and damage control surgery (DCS).

Permissive hypotension, as discussed, prevents further bleeding from recently clotted vessels. Hemostatic resuscitation involves early use of blood and blood products to minimize coagulopathy, prevent dilutional coagulopathy, and improve survival [20, 21]. It entails the use of plasma, platelets, and red blood cells in an optimal ratio of 1:1:1 as well as the use of antifibrinolytic agents such as tranexamic acid in addition to limiting the use of crystalloids [22]. Hemostatic monitoring is applied so that when bleeding slows, a goal-directed approach for resuscitation can be adopted [22]. Massive transfusion protocol (MTP) should be activated in patients requiring continued resuscitation [3] and started as early as possible to avoid rapid administration of crystalloids [23] and post-injury complications such as organ failure and abdominal compartment syndrome [24].

DCS restores physiology instead of providing definitive anatomical repair. It consists of bleeding control, decontamination, quick body cavity closure to rewarm the patient, and planned re-operation for definitive repair [20].

## Ideal resuscitation fluid

Though crystalloids and colloids are widely used for fluid resuscitation, the ideal choice of fluid is debated. Hypotonic fluids do not stay intravascular. Therefore, isotonic and hypertonic crystalloids are used for fluid resuscitation. Lactated Ringer’s (LR) or normal saline (NS) is the primary resuscitation fluids [18]. Albumin and gelatin solutions are protein colloids whereas starches and dextrans are non-protein colloids.

## Crystalloid versus colloid debate

The Saline versus Albumin Fluid Evaluation (SAFE) study compared 4% albumin and NS. Both showed clinically equivalent efficacy. The volume of fluid administered was less with albumin than with NS (1:1.4) [25]. However, in TBI patients, albumin resuscitation was associated with higher mortality compared to NS [26]. In trauma patients who required > 10 units of packed red blood cells and underwent DCS, permissive hypotension with hypertonic saline (HTS) involved less fluid requirements, reduced 30-day mortality, increased urine output, and reduced risk of acute respiratory distress syndrome, sepsis, and organ failure compared to standard resuscitation with isotonic crystalloids. However, there was no difference in renal failure [27].

A multicenter randomized controlled trial called the Colloids Versus Crystalloids for the Resuscitation of the Critically Ill (CRISTAL) trial compared mortality in critically ill patients who received colloids (*n* = 1414; gelatins, dextrans, hydroxyethyl starches, or 4% or 20% of albumin) or crystalloids (*n* = 1443; isotonic or hypertonic saline or LR solution) for fluid resuscitation [28]. Therapy was open-label but the outcome assessment was blinded to treatment assignment. There was no difference in 28-day mortality, need for renal replacement therapy, development of organ failure, and number of hospital days between the two groups [28]. The 90-day mortality was slightly lower with colloids. This needs further evaluation [28].

In the Crystalloid versus Hydroxyethyl Starch Trial (CHEST), hydroxyethyl starch (HES) was associated with increased renal failure, need for renal-replacement therapy [29], and mortality [30]. However, risks of renal injury and mortality related to colloids were observed only in critically ill patients with sepsis [31].

## Recommendations

Crystalloids are generally readily available and inexpensive [32]. They are preferred in TBI [26] and in initial resuscitation of trauma patients [18]. Compared to non-balanced fluids, L-isomer of LR causes less inflammation, immune dysfunction [33], and mortality in critically ill patients [34] and is recommended fluid of choice in hemorrhagic shock patients [18]. HTS is beneficial in patients with brain edema [35], TBI [36], or massive hemorrhage requiring DCS [27]. Though HTS contributes to renal failure, it significantly decreases the fluid requirement and consequent acute respiratory distress syndrome related to interstitial fluid overload [27, 35]. Chloride-restrictive fluids reduce the risk of renal failure and the need for renal replacement therapy [37]. They may be used as adjuncts to blood products and other therapies.

Colloids remain intravascular longer, rapidly expand plasma volume, and achieve similar goals quickly with less volume than crystalloids. However, they come with added expense and lack of survival benefit over crystalloids [38]. Colloid use is recommended when patients cannot tolerate large crystalloid volumes and overload is of concern [25]. Albumin is contraindicated in TBI [26], and HES and other starches are not recommended [29–31]. Owing to the increased risk of kidney injury, colloids should be cautiously used in patients with renal impairment. Renal effects are colloid-specific; albumin displays renoprotection while HES shows nephrotoxicity [39].

## Advantages of new generation gelatins

Gelatins are low molecular weight, cheaper than albumin and other synthetic colloids, rapidly excreted by kidneys, associated with less renal impairment than HES, and have no upper limit of volume that can be infused unlike starches and dextran [40]. They have a lower risk of dilutional coagulopathy than dextrans and starches [41]. Though gelatins are associated more with anaphylactoid reactions than albumin, some recent studies showed no anaphylactic reactions with polygeline [42, 43]. New generation gelatins may have a significant role in remote/rural settings to prevent crystalloid overuse until definitive care is available and also in low-income settings where albumin may not be available/affordable. In India, polygeline is routinely used in hypovolemic trauma patients. Polygeline has a short half-life of 4–6 h and is readily excreted in the urine and does not seem to adversely affect renal function [42]. It does not accumulate in patients with renal failure [43]. Though polygeline seems to be safe and effective to treat hypovolemic trauma patients [42, 43], further large and multicenter comparative studies are warranted to validate results and elucidate outcome benefits.

## Post-resuscitation fluid management

Both under-resuscitation and over-resuscitation are fatal. Transitioning from the initial resuscitation to post-resuscitation phase is important to improve outcomes. The post-resuscitation phase is the period after coagulopathy is corrected, microcirculatory flow is improved, and hemodynamic parameters are stabilized (SBP > 100 mmHg; MAP > 65 mmHg in most cases) [15]. During this phase, markers for fluid responsiveness are normalized. Patients are no longer fluid responsive. If a patient is fluid responsive but the risks of fluid challenge outweigh the hemodynamic benefits, then other options should be considered such as inotropic support and hemodialysis with net ultrafiltration. Awareness of Resuscitation, Organ support, Stabilization, Evacuation (ROSE) concept [15] is essential for timely and appropriate decisions. Maintenance fluids should be given such that they stay intravascular for longer periods to avoid tissue edema. During this phase, crystalloids are used for both supplementing the fluid and administering medicines. Fluids used for administering medicines and supplementing nutrition together should not exceed 2 mL/kg/h [15]. Balanced fluids are better than 0.9% NS especially if sodium and chloride overload is of concern [15].

## Resuscitation in special groups

### Pediatrics

In children, isotonic and balanced crystalloid (20 mL/kg) is recommended for initial resuscitation. Clear fluid volume should be < 40 mL/kg to prevent dilutional coagulopathy and edema [44]. During the maintenance phase, children are prone to hyponatremia and cerebral edema if hypotonic solutions are administered excessively [45]. So, limited volumes (maximum 2 mL/kg/h) using flow controllers are recommended [15].

### Geriatrics

Aging causes arterial stiffness and decreased left ventricle (LV) compliance. Hypovolemia decreases preload leading to under-filling of ventricles with disproportionate drop in cardiac output [15]. Therefore, permissive hypotension should be applied cautiously with adequate monitoring [15]. Similarly, hypervolemia increases the risk of pulmonary edema due to decreased LV compliance [15]. Echocardiography is recommended to assess fluid requirements [15]. Clear fluid should be limited to 20 mL/kg, blood and blood products administered early, and hemoglobin levels > 9 g/dL and MAP > 70 mmHg maintained [15].

### Burns

Parkland formula [fluid requirement = total body surface area (TBSA, %) × 4 mL × body weight (kg)] used for fluid resuscitation in burn patients does not compensate for depth [46]. Deeper and extensive burns require more fluid which increases edema and morbidity [47]. Therefore, in clinical settings, fluid requirements are usually 5 mL/kg/%TBSA during initial 24 h [48]. Around 50% of the daily fluid requirement is given in initial 6 h [15]. LR is preferred [48], while hyper-oncotic colloids may cause acute kidney injury (AKI) [15, 49].

Enteral resuscitation is started during the initial 6 h [15]. Oral resuscitation works better for burns < 15%TBSA [48]. For enteral feeding, standard formula with 2 mL/kg/h may be used with a further increase every 3 h till the goal rate calculated for the patient is attained [50]. Patient hematocrit should be below 40% within the initial 6 h and urine output should be maintained at around 1 mL/kg/h [15].

In pregnant patients with burns, both Parkland formula and clinical signs such as vital signs, urine output, and fetal heart rate are considered to prevent under-resuscitation because the intravascular volume is increased during pregnancy [46].

### Pregnancy

Pregnant patients tolerate blood loss better due to increased circulating blood volume and cardiac output [46, 51]. Supplemental oxygen should be provided to prevent maternal and fetal hypoxia. Adequate volume replacement is also necessary for adequate uteroplacental blood flow [46]. Absence of tachycardia and hypotension should not be considered as the absence of significant hemorrhage because those signs usually occur in pregnant women after 1500–2000 mL of hemorrhage [46]. The fetal heart rate is sensitive to maternal hypovolemia and should be monitored [46].

### Chronic kidney disease

Both fluid overload and fluid composition affect the kidneys. Compared to buffered crystalloids, isotonic saline reduces renal perfusion and increases the risk of AKI [52]. Balanced electrolytes cause less hyperchloremia and are preferred. NS may cause kidney injury and increase acidosis [2]. Due to the risk of kidney injury, chloride-liberal fluids should be restricted [37] and colloids should be used cautiously [31, 52, 53].

### LV failure

Excessive fluid administration in patients with decreased LV compliance worsens lung congestion and non-cardiogenic pulmonary edema resulting in pulmonary hypertension, right ventricle dysfunction, and further decrease in LV volumes [54]. Echocardiography is recommended to assess cardiac load and cardiac response to fluid administration [54]. In patients with life-threatening hypotension, both vasopressors and fluids should be given to maintain target arterial pressure [2]. Whenever cardiac output monitoring is not available and a patient is not responding to fluid challenge/norepinephrine, cardiac dysfunction should be suspected and treated accordingly [2].

### Alcoholic liver disease

Cirrhotic patients have elevated cardiac output, decreased systemic vascular resistance, and low BP [55]. This is due to total extracellular fluid overload while there is central effective circulatory hypovolemia.

In trauma patients with cirrhosis, fluid loading may be needed. However, the fluid load may worsen organ function and contribute to ascites. Therapeutic paracentesis is recommended in patients with tense ascites and MAP ≥ 60 mmHg is appropriate in cirrhotic patients [56]. Pulmonary artery catheter or echocardiography should be used to monitor fluid overload [56]. In volume-depleted patients, crystalloids are the initial fluid of choice (10–20 mL/kg). Balanced salt solutions are preferred in hyperchloremic acidic patients [56]. Albumin should be administered following large-volume paracentesis (> 5 L) as it prevents post-paracentesis circulatory dysfunction better than crystalloids [55]. HES is contraindicated due to nephrotoxicity.

### Massive blood loss

In patients with massive blood loss, permissive hypotension prevents progression to dilutional coagulopathy of trauma [57]. In severe and uncontrolled hemorrhagic shock, controlled resuscitation (MAP of 40 mmHg) is preferred [58]. International guidelines recommend SBP of 80–90 mmHg in trauma without brain injury and MAP ≥ 80 mmHg in TBI until major bleeding is controlled [2].

New generation gelatins like polygeline may maintain circulation until blood is available. Improvements in BP, MAP, pulse rate, respiratory rate, and blood pH are noted within 1 h of administration in hypovolemic trauma patients with sustained benefits even after 24 h [42, 43, 59].

## Conclusions

Fluid resuscitation strategies have evolved with time. Different traumas need different fluids and different resuscitation strategies. Prehospital trauma care reduces mortality in rural/remote settings. Delayed fluid resuscitation is preferred when transport time to definitive care is shorter whereas goal-directed resuscitation with low-volume crystalloid is preferred if transport time is longer. Adhering to evidence-based clinical practice guidelines and local modifications based on patient population, available resources, and expertise may improve patient outcomes.

## Data Availability

NA

## Abbreviations

AKI: Acute kidney injury
BP: Blood pressure
CHEST: Crystalloid versus Hydroxyethyl Starch
CRISTAL: Therapy in the Colloids Versus Crystalloids for the Resuscitation of the Critically ill
DCS: Damage control surgery
HES: Hydroxyethyl starch
HTS: Hypertonic saline
LR: Lactated Ringer’s
LV: Left ventricle
MAP: Mean arterial pressure
MTP: Massive transfusion protocol
NS: Normal saline
PTC: Prehospital trauma care
PTT: Prehospital transport time
SBP: Systolic blood pressure
SV: Stroke volume
TBI: Traumatic brain injury
TBSA: Total body surface area
